# Metabolomic Approaches for Detection and Identification of Biomarkers and Altered Pathways in Bladder Cancer

**DOI:** 10.3390/ijms23084173

**Published:** 2022-04-10

**Authors:** Nicola Antonio di Meo, Davide Loizzo, Savio Domenico Pandolfo, Riccardo Autorino, Matteo Ferro, Camillo Porta, Alessandro Stella, Cinzia Bizzoca, Leonardo Vincenti, Felice Crocetto, Octavian Sabin Tataru, Monica Rutigliano, Michele Battaglia, Pasquale Ditonno, Giuseppe Lucarelli

**Affiliations:** 1 Department of Emergency and Organ Transplantation-Urology, Andrology and Kidney Transplantation Unit, University of Bari, 70124 Bari, Italy; nickant.dimeo@gmail.com (N.A.d.M.); d.loizzo27@gmail.com (D.L.); monica.rutigliano@virgilio.it (M.R.); michele.battaglia@uniba.it (M.B.); pasquale.ditonno@uniba.it (P.D.); 2 Division of Urology, Virginia Commonwealth University (VCU) Health, Richmond, VA 23298, USA; pandolfosavio@gmail.com (S.D.P.); riccardo.autorino@vcuhealth.org (R.A.); 3 Division of Urology, University of Naples “Federico II”, 80100 Naples, Italy; 4 Division of Urology, European Institute of Oncology (IEO), IRCCS, 20141 Milan, Italy; matteo.ferro@ieo.it; 5 Department of Biomedical Sciences and Human Oncology, University of Bari, 70124 Bari, Italy; camillo.porta@uniba.it (C.P.); alessandro.stella@uniba.it (A.S.); 6 Department of General Surgery “Ospedaliera”, Polyclinic Hospital of Bari, 70124 Bari, Italy; cinziabiz84@gmail.com (C.B.); dr.leonardo.vincenti@gmail.com (L.V.); 7 Department of Neurosciences, Reproductive Sciences and Odontostomatology, University of Naples “Federico II”, 80131 Naples, Italy; felice.crocetto@unina.it; 8 I.O.S.U.D., George Emil Palade University of Medicine and Pharmacy, Science and Technology, 540142 Targu Mures, Romania; sabin.tataru@gmail.com

**Keywords:** bladder cancer, metabolomics, biomarker

## Abstract

Metabolomic analysis has proven to be a useful tool in biomarker discovery and the molecular classification of cancers. In order to find new biomarkers, and to better understand its pathological behavior, bladder cancer also has been studied using a metabolomics approach. In this article, we review the literature on metabolomic studies of bladder cancer, focusing on the different available samples (urine, blood, tissue samples) used to perform the studies and their relative findings. Moreover, the multi-omic approach in bladder cancer research has found novel insights into its metabolic behavior, providing excellent start-points for new diagnostic and therapeutic strategies. Metabolomics data analysis can lead to the discovery of a “signature pathway” associated with the progression of bladder cancer; this aspect could be potentially valuable in predictions of clinical outcomes and the introduction of new treatments. However, further studies are needed to give stronger evidence and to make these tools feasible for use in clinical practice.

## 1. Introduction

Bladder cancer (BCa) is the tenth most-common malignant tumor of the urinary tract, accounting for 549,000 new cases and about 200,000 deaths per year worldwide [1]. Risk factors for BCa include age, sex, race, smoking, occupational exposure to aromatic amines, polycyclic aromatic hydrocarbons and chlorinated hydrocarbons, socioeconomic status, diet, and pathogen infections [2,3,4,5,6,7]. However, the pathogenesis of BCa and its stage-wise progression have not yet been fully explained and defined. [8] BCa can be classified as low-grade (LG) or high-grade (HG), according to its histopathological characteristics [9,10]. According to the invasiveness of the tumor, BCa has traditionally been classified as a non-muscle-invasive bladder cancer (NMIBC) or a muscle-invasive bladder cancer (MIBC). NMIBC is the most frequently diagnosed and is associated with high recurrence rates, whilst MIBC has a high rate of metastasis and a 5-year survival rate of 63% for T2, 46% for T3, and 15% for T4 tumors [9,10,11]. However, same-grade BCa may have different prognoses and responses to treatment, which can be a reflection of molecular heterogeneity among histologically similar tumors [12,13,14]. Advances in molecular pathology have driven efforts to classify BCa into subtypes, both to understand the molecular heterogeneity and to facilitate clinical decision making regarding systemic therapies through the identification of prognostic and predictive markers [14,15]. In recent years, a metabolomics approach has been implemented to study the tumorigenesis of different cancers, focusing on pathogenesis and biomarkers research [16,17,18,19,20,21]. Moreover, it contributed to understanding the relevant alterations of the catabolic and anabolic processes that are impaired in cancer cells [22,23]. In this review, we discuss the role of metabolomics in diagnostic management and BCa pathologic profiling.

## 2. Molecular Classification of Bladder Cancer

NMIBC and MIBC have different clinical behaviors; therefore, histological and cytogenetic analysis revealed two distinct carcinogenic pathways: the papillary and the non-papillary pathway. The two-pathway theory is used to explain early-phase disease, but given the complexity and high heterogeneity of BCa, it cannot explain the biology and the progression of tumors. The development of advanced techniques, such as sequencing and mass spectrometry (MS), has provided better tumor profiling [24,25,26,27], allowing for the identification of molecular subtypes with distinct clinical behaviors. Several molecular classifications have been defined; however, there are significant subtype-related differences among definitions, classification methods, oncological outcomes, and responses to cancer treatment [13,14,28,29]. BCa molecular subtypes in the literature share different molecular features; two to seven distinct subtypes have, nevertheless, been identified [13,30,31,32].

In 2014, Damrauer et al., examining data from 262 BCa samples, evidenced the basal-like and luminal subtypes of HG BCa, reflecting the hallmarks of breast cancer biology [30]. Sjödahl and colleagues identified five different subtypes: urobasal A (UroA), genomically unstable, urobasal B (Uro B), squamous cell carcinoma-like, and infiltrated [33]. Hedegaard et al. identified three classes of NMIBC molecular subtypes [34]. Class I expresses elevated levels of early cell-cycle regulators (CCDN1) and FGFR3 (fibroblast growth factor receptor 3) mutations. This subtype is largely represented in low-grade, non-invasive tumors associated with a good prognosis, showing luminal-like features that are comparable to the urobasal A subtype (UroA). Class II has an increased expression of late cell-cycle regulators and transcription factors associated with epithelial-to-mesenchymal transition (EMT) and ERBB2. It presents luminal-like features and is comparable to the genomic unstable (GU) subtype, as described by Sjödahl and colleagues. Class III is associated with basal-like phenotype and FGFR3 mutations [35].

Several groups have also proposed comparable MIBC molecular classifications, which have been associated with specific biological features, clinical outcomes, and therapy effectivenesses [14,29,30,31,36,37,38,39,40,41]. A consensus classification has been introduced to better handle molecular subtypes in clinical trials and in clinical practice [13]. This consensus classification derives from the combination of the analysis of six previously published classifications of MIBC with public transcriptome data [35]. This resulted in the description of six molecular subtypes for MIBC (Table 1): luminal papillary (LumP), luminal non-specified (LumNS), luminal unstable (LumU), stoma-rich, basal/squamous (Ba/Sq), and neuroendocrine-like (NE-like) [14]. BCa molecular classifications are strong predictors of oncological outcomes and responses to treatment. However, due to significant heterogeneity among studies in the literature, a consensus based on well-designed prospective studies is mandatory to improve BCa management [13,42].

## 3. Metabolomics and Bladder Cancer

Metabolites are the end-products or the downstream, intermediate, low-molecular weight products of metabolic pathways (glucose, lipids, amino acids, nucleotide metabolites). Metabolomics is defined as the study of metabolites in a biological sample (e.g., urine, blood) involved in the regulation of catabolic and anabolic pathways under a certain physiological or pathological condition [43]. Metabolomic analyses measure molecules ranging in polarity from water-soluble acids to non-polar lipids. These molecules have disparate physical properties [44] and are, resultingly, less complex than genomics, transcriptomics, and proteomics. This new field investigates cancer metabolism to better understand cancer-related changes, improving the identification of tumor-specific metabolic biomarkers with a potential diagnostic, prognostic, or predictive value [33]. This approach has been already applied to several cancers, including breast [45], ovary [46], kidney [47,48], prostate, [49] colorectum [50], and hepatocellular carcinomas [51], through the screening and detection of metabolite levels in human biofluids [52]. The identification of metabolites in a biological sample requires analytical approaches such as MS or nuclear magnetic resonance (NMR) spectrometry [53].

The MS-based methodology offers a quantitative analysis of metabolites with high selectivity, sensitivity, and accuracy [53,54,55]. This technique usually needs to be coupled with a separation method, and metabolites are identified based on their retention times through this process. To date, gas chromatography (GC), high-performance liquid chromatography (HPLC), and capillary electrophoresis (CE) are the most common techniques employed for the segregation of metabolites, in addition to NMR-MS, which exploits the magnetic resonant frequencies of nuclei (Table 2) [56,57]. These methods have become critical techniques for quantitatively and qualitatively measuring the metabolome through the creation of large-scale informatics datasets [58,59,60].

Metabolomics could be useful in BCa research to identify potential non-invasive and highly sensitive biomarkers. Profiling the global BCa metabolome using MS and NMR has led to the discovery of several metabolites [12]. These are involved in biochemical pathways including glycolysis, the tricarboxylic acid (TCA) cycle, fatty acid β-oxidation, and amino acid metabolism. To date, the heterogeneity of samples (blood, urine, tumor cell lines) offers available results in different approaches to this field.

### 3.1. Urine Samples

Urine is a useful sample in bladder pathology [61,62]. Therefore, urine metabolomic characteristics may closely reflect the state of the bladder in different physiological and pathological conditions (BCa, interstitial cystitis) [56,58,59,60,63]. Furthermore, urine has always been thought as a diagnostic or prognostic instrument, increasing the search for new biomarkers in this field and becoming a promising strategy in BCa detection.

Pasikanti et al. [64] have been pioneers in the investigation of the role of urinary metabolomics in BCa. They identified 15 metabolite patterns that are useful for characterizing NMIBC patients. Metabolic analyses showed that many metabolites involve the tyrosine and tryptophan pathways, suggesting that amino acid metabolism might play a role in the pathogenesis of BCa. They observed a 100% sensitivity in BCa detection, compared to the traditional cytology approach, which had a sensitivity of only 33%. Additionally, urinary metabolomics exhibited feasibility in the staging and grading of bladder tumors. In 2011, Putlury at et al. [65] found 25 altered-urine metabolites in cancer patients, compared with control urine from individuals having no history of BCa. They designed a partial least squares discriminant analysis (PLS-DA)-based classification model for detecting bladder cancer in urine, with an overall accuracy between 67 and 72% in four independent cohorts. The results were encouraging, considering that benign controls of two cohorts were from individuals with previous BCa who were in remission following immuno-/chemo-therapeutic treatment. Shen et al. [66] studied the potential role of metabolomics in BCa early detection. Urine samples from 23 early stage BCa patients and 21 healthy volunteers were analyzed with the ultra-performance liquid chromatography–high-resolution mass spectrometry (UPLC-HRMS) method. They identified over 9000 UPLC-HRMS features, and a sample analysis showed 3 BCa-upregulated metabolites: nicotinuric acid, trehalose, and aspartate–aspartate–glycine–tryptophan tetrapeptide. Nicotinuric acid is an endogenous end-product of the nicotinate and nicotinamide metabolism, and a minor metabolite of fatty acid β-oxidation. Trehalose is a non-reducing sugar with antioxidant properties, usually found in extracellular space. There were also three BCa down-regulated metabolites: inosinic acid, involved in purine metabolism; ureidosuccinic acid, an intermediary product in both aspartate and pyrimidine synthesis; and glycine–cysteine–alanine–lysine tetrapeptide. Receiver operating characteristic (ROC) analyses, using two linear regression models, showed a high diagnostic performance for detecting BCa with area under the curve (AUC) values between 0.919 and 0.934. The researchers analyzed six urine samples collected seven days after a transurethral resection of bladder tumor (TURBT) procedure, which showed significant changes in glycine–cysteine–alanine–lysine nicotinic acid and inosinic acid compared with preoperative values. These restorative changes indicate that these metabolite markers are directly related to BCa.

Cheng et al. [67] conducted a LC-HRMS metabolomic profile, aiming to discover biomarkers for the detection of BC at early stages. A total of 284 subjects (117 healthy adults vs. 80 NMIBC patients without hematuria, and 87 patients with hematuria) were enrolled. The metabolite panel, including dopamine 4-sulfate, aspartyl–histidine, and tyrosyl–methionine, was found in a discovery set. The predictive ability to distinguish the NMIBC group from the control group had an AUC of 0.838 in an external validation set. The AUC of the panel for LG-NMIBC samples, which consisted of 3-hydroxy-cis-5-tetradecenoylcarnitine, 6-ketoestriol, β-cortolone, tetrahydrocorticosterone, and heptylmalonic acid, was 0.899. The sensitivity and specificity were 0.881 and 0.786, respectively. Jin et al. [20] analyzed the urinary metabolomic profiles of 138 patients with BCa and 121 control subjects using high-performance liquid chromatography–quadrupole time-of-flight mass spectrometry (HPLC-QTOFMS). A multivariate statistical analysis revealed differences between MIBC and NMIBC patients. Successive analyses identified 12 differential metabolites involved in glycolysis and β-oxidation, which contributed to the distinction between the BCa and control groups. The cancer group had elevated levels of urinary acetyl-CoA, carnitine, and acylcarnitine. The association of these metabolites with cancer was corroborated by previous microarray results, showing that carnitine transferase and pyruvate dehydrogenase complex expressions are significantly altered in cancer groups. In terms of clinical applicability, the model diagnosed BCa with a sensitivity and specificity of 91.3% and 92.5%, respectively, with comparable results obtained by ROC analysis (AUC = 0.937). Multivariate regression also suggested that the metabolomic profile correlates with cancer-specific survival time.

In 2018, Loras et al. [68] evaluated metabolomic profiling as a predictor of recurrence in BCa surveillance. In this study, they examined changes in the urinary metabolome of 316 urine samples collected from 31 NMBIC patients before and after TURBT. The discriminant analysis of UPLC-MS metabolic profiles displayed high sensitivity (87.9%) and specificity (100%). The negative predictive values for low, low–intermediate, high–intermediate, and high-risk patient groups were 96.5%, 94.0%, 92.9%, and 76.1%, respectively.

The study revealed that an altered phenylalanine, arginine, proline, and tryptophan metabolism was associated with NIMBC. A pilot retrospective analysis of BCa metabolic biomarkers during post-TURBT surveillance was carried out: the results showed a gradual shift in the metabolic profile towards the BCa profile when the tumor reappeared, as confirmed by cystoscopy. Furthermore, external factors such as diet, water consumption, environmental exposure, or drug intake may affect the urine metabolome, resulting in potential confounding bias [69,70].

Liu et al. [71] investigated sex-related and age-related variability in urine samples from 203 healthy volunteers, 6 patients with non-malignant bladder pathologies, and 53 patients with BCa. An inter-individual analysis of both healthy controls and BCa patients showed that urine metabolome was relatively stable. The median of the interindividual coefficient of variation (CV) was 0.94; the inter-individual variation was similar in female (0.891) and male (0.890) urinary profiles. Similar to the normal urine metabolome, the CV in females and males with BCa was smaller, suggesting that sex contributes to BCa urine metabolome variation. Further analysis showed that tryptophan metabolism, the citrate cycle, and pantothenate and CoA biosynthesis were also related to sex and age. To avoid age and sex interference, additional BCa urine metabolomic biomarkers were explored using age- and sex-matched urine samples. A metabolite panel was discovered to have good predictive ability for BCa: the panel includes trans-2-dodecenoylcarnitine, serinyl-valine, feruloyl-2 hydroxyputrescine, and 3-hydroxynonanoyl carnitine with an AUC of 0.956. Further studies were conducted by Jacyna et al. [72], who matched BCa and control groups in terms of risk factors such as obesity, age, gender, and tobacco smoking to exclude their influence on differences in urine metabolomics profiles.

Hematuria, known as one of symptoms of BCa, may also occur in physiological and other pathological conditions (menstruation, urinary tract infections). For this reason, it is considered a confounding factor in terms of the presence of hemoglobin in urine samples. Metabolites of hemoglobin in urine might influence the diagnostic value of urine metabolomics as a biomarker for BCa. The knowledge of these pathological and gender-related perturbations must be controlled when designing studies to obtain useful diagnostic information. Cheng et al. [67] and Jin al et al. [20] considered this aspect in their respective studies, evaluating differences in NMIBC groups with or without hematuria. The researchers, using multivariate statistical analysis, found that the confounding factor of hematuria can be well removed.

### 3.2. Blood Serum Samples

Several works have been made in order to detect serum metabolites, proteins, or inflammatory cells to be employed as biomarkers in early cancer detection and surveillance [73,74,75,76,77,78,79,80,81]. In 2012, Cao et al. [82] performed 1H-NMR-based metabolomic analyses on serum samples from three groups: BCa patients, calculi patients, and healthy controls. The orthogonal partial least squares discriminant analysis (OPLS-DA) showed that the serum metabolic profiles from BCa patients had decreased levels of isoleucine/leucine, tyrosine, lactate, glycine, citrate, as well as increased levels of very-low-density lipoproteins (VLDL), glucose, and acetoacetate. The metabolite levels in post-TURBT BCa patients were somewhat recovered to those in healthy subjects. Furthermore, the alteration trends in glucose, tyrosine, and phenylalanine levels were closely related to the status of metastasis, suggesting a potential role in surveillance for invasive cancer progression. Lin et al. conducted a LC-MS-based serum profiling study, including 24 BCa patients, 24 kidney cancer (KC) patients, and 24 healthy control patients [83]. The results showed six and five metabolites as specific biomarkers for BCa and KC, respectively, and seven metabolites as common biomarkers for both BCa and KC. The findings were evaluated with ROC analysis: eicosatrienol and azaprostanoic acid had the highest AUC (0.980 and 0.977) of all BCa-specific biormarkers. Acetylphenylalanine and methyl hippuric acid had the highest AUC (0.847 and 0.828) among the common genitourinary biomarkers.

Liu et al. [84] carried out a similar work, enrolling 64 BCa patients, 74 KC patients, and 141 healthy controls in order to discriminate their global plasma profiles. Metabolomic and lipidomic analyses showed eight common differential metabolites with good predictive value for BCa, KC, and control plasma sample discrimination. Homocysteine thiolactone, acetylcysteine, methionine sulfoximine, 9,10,13-TriHOME, avenoleic acid, (10E,12Z)-(9S)-9-Hydroperoxyoctadeca-10,12-dienoic acid, and 16-Hydroxy-10-oxohexadecanoic acid were down-regulated in cancer groups compared to the control group. In addition, the relative content in the KC group was also lower than that in the BCa group. Moreover, 9S,10R-Epoxy-6Z-nonadecene was up-regulated in the cancer groups compared with the control group, and the relative content in the KC group was lower than that in the BCa group. Further studies evaluated the prediction of HG-BCa development. Bansal et al. [85] demonstrated that the combination of dimethylamine (DMA), glutamine, and malonate could accurately differentiate LG BCa from HG BCa using 1H NMR-derived serum data. Using OPLS-DA, they found that the combination had 96% sensitivity and 94% specificity using an external validation set. These findings highlighted the potential use of NMR-derived serum metabolomics in the BCa grade categorization. Differences in energy metabolism within LG/HG BCa and health control samples were also found by Zhou et al. [86]. They performed a pseudo-targeted metabolomics procedure based on GC-MS with selected ion monitoring (GC-MS-SIM), observing that metabolites specific to the pentose phosphate pathway (PPP), nucleotide, and fatty acid synthesis were increased in LG/HG BCa, compared to the healthy control, with an AUC of 0.863.

In 2016, Tan et al. [87], through UHPLC-QTOFMS, analyzed LG vs. HG BCa metabolomic serum profiles. They identified 11 metabolites related to different functions in cancer progression, such as tumor cell proliferation, immune escape, differentiation, apoptosis, and cell invasion. Three metabolites, including inosine, N1-acetyl-N2-formyl-5-methoxykynuramine, and phosphatidylserine, were selected to evaluate a potential diagnostic performance. ROC analyses on the prediction model showed an AUC of 0.961 (sensitivity was 88.2% and specificity was 91.2%). Further studies evidenced racial disparities in BCa development. Vantaku et al. [88] identified 53 metabolites in serum samples, mainly related to the amino acid, lipid, and nucleotide metabolism, which showed significant differences between African American (AA) and European American (EA) patients. Using LC-MS, they characterized the metabolome of either AA BCa or EA BCa. It has been shown that levels of taurine, glutamine, glutamate, aspartate, and serine were elevated in serum samples of AA patients when compared with EA patients, and levels of the purine catabolic pathway metabolites hypoxanthine and uric acid were higher in AA patients as well.

### 3.3. Tissue Samples

To the best of our knowledge, the literature lacks strong evidence for the metabolomic signature of BCa tissue samples. Putluri et al. [65] conducted a multi-center study through a LC-MS-based analysis of 58 BCa tissue samples (27 benign-adjacent, 31 BCa, 25 matched pairs). Exploratory analyses identified 35 significant metabolites. Elevated levels of aliphatic amino acids, namely, serine, asparagine, and valine, or their aromatic counterparts, such as tryptophan, phenylalanine, and histidine, were found among the perturbed metabolites. Similarly, in 2013, Tripathi et al. [89] carried out a 1H-HRMS-NMR-based metabolomic analysis of 59 benign and BCa tissues. The results identified 22 clearly differentiated metabolites in BCa samples, compared with either benign or healthy ones. These results were cross-validated via targeted GC-MS analysis, demonstrating the potential of these biomarkers in clinical diagnosis. BCa tissues were characterized with decreased triglyceride levels and increased levels of branched-chain amino acids, lactate, alanine, acetate, glutamic acid, glutathione, glutamine, aspartate, creatine, choline-containing compounds (choline, phosphocholine and glycerophosphocholine), taurine, myo-inositol, phenylalanine, and tyrosine. Noticeably, striking metabolic signatures were observed even for early stage BCa tissues (Ta-T1), demonstrating the sensitivity in detecting BCa.

In 2017, Piyarathna et al. [90] analyzed 165 bladder-derived tissues (126 BCas, 39 benign-adjacent or normal) in order to identify the lipidomic signatures associated with the survival and different clinical stages of BCa. Using LC-MS profiling, they found significant alteration in lipids such as phosphocholines, phosphatidylethanolamines (PE), plasmenyl PEs (up-regulated), and TGs (down-regulated). The panel of altered lipids was mapped to their corresponding pathway’s genes using the Human Metabolic Database, resulting in the identification of 59 genes. The expression of PE lipids was higher in male than in female patients with BCa, the expression of diglycerides was higher in tumors from LVI-positive patients, and the expression of lyso-PE lipids was higher in patients with any positive node or Nx, compared to those with N0. A further study [91] has been conducted to explore metabolomic changes in BCa patients treated with submucosal injection of gemcitabine followed by TURBT. This study examined 48 BCa tissue samples treated with gemcitabine, as well as adjacent normal tissues from 12 of those patients. Based on HPLC-Q-Exactive-MS analysis, they found 34 significantly altered metabolites associated with BCa. Three metabolic pathways, namely, glutathione, purine, and thiamine, were altered in BCa, with the glutathione metabolism being the most-altered pathway. Moreover, the levels of bilirubin and retinal were recovered after gemcitabine injection, suggesting that these two molecules are likely the targets of gemcitabine treatment.

### 3.4. Cell Lines

Studies have also focused on in vitro approaches to obtain further information about metabolic pathways which could lead to BCa carcinogenesis and progression.

In vitro cell culture represents a less-complex disease model, and has many advantages over other approaches, including simpler and controllable settings and less variability among samples [12,92,93,94,95]. Dettmer et al. [96] first investigated cancer cells’ metabolome by means of LC-Q-TOF MS analysis of 20 different human cancer cell lines, including BCa cell lines J82 and RT4, in order to define a common metabolic foot-printing. They observed that high lactate production was a common expected feature of all neoplastic cells, as the most discriminative features between different cell clusters were various di- and tripeptides (valine–leucine and leucine–leucine). A relationship between changes in the glycolytic profile and BCa progression was studied through an NMR-based analysis of RT4 and TCCSUP cell lines by Conde et al. [97]. They observed an increased pyruvate consumption, consistent with the higher levels of lactate and alanine production, in highly invasive cell lines. Similar results were found by Petrella et al. [98]. They characterized the nutrients’ intake and the products’ excretion in the extracellular medium of three BC cell lines of different grades of malignancy. NMR-MS analysis results showed that low-grade cells had an increased consumption of arginine, glutamine, branched-chain amino acids (BCAAs), and serine, along with an increased excretion of formate. All these compounds suggested a strong relationship with an active oxidative metabolism. On the other hand, alanine excretion was higher in RT4 (LG), showing a very clear trend associated with different grades of malignancy. Extracellular pyruvate levels in 5637 cells (HG) were lower than RT4 ones, with a reverse proportion in lactate levels.

Rodrigues et al. [99] conducted a GC-MS study to discriminate metabolomic differences between LG and HG BCa cultured cell lines. They found, in HG cells, significantly lower levels of FAs, including myristic, palmitic, and palmitoleic acids, compared to LG BCa cells. Furthermore, they observed significantly altered levels of amino acids between long- and high-grade BCa, namely, glycine, leucine, methionine, valine, and aspartic acid. These results clearly demonstrated that advanced grades require more FA consumption for survival and continuous growth. A further NMR-MS study conducted by Iliou et al. [100] investigated four BCa cell lines which differed in malignancy and aggressiveness. The different energetic metabolisms have been confirmed, as AMP and creatine phosphate were highly increased and in grade-III cell lines compared to grade-I ones. New perspectives in specific biomarkers research have been rising with cell lines studies through GC-MS-based analyses of volatile organic compounds (VOCs). Rodrigues et al. [101] compared VOCs from the culture media of BCa to VOCs from normal cell lines: they revealed distinct metabolite patterns as potential diagnostic biomarkers for BCa, based on the level of 2-pentadecanone, dodecanal, and gamma-dodecalactone. Recently, Pinto and colleagues have shown the VOCs’ profile from a BCa patient’s urine samples [102]. Compared to a healthy control cohort, they have demonstrated that BCa patients have higher levels of alkanes and aromatic compounds (1-methylnaphthalene, 2-methylnaphthalene, 1,2,4-trimethylbenzene, and p-cresol). Moreover, this was associated with lower levels of aldehydes, ketones, and monoterpenes, disclosing 70% sensitivity, 89% specificity, and 80% accuracy. Different urinary volatile profiles were also found among patients diagnosed at different stages, suggesting a potential role of VOCs in prognostic assessment and stratification (Supplementary Table S1).

## 4. Multi-omics Integration and Metabolic Pathways

BCa metabolomic profiling is usually the first step in the definition of specific cancer-related pathways. Thanks to the integration of metabolomic and transcriptomic information, altered metabolites and lipids have been linked to their corresponding genes. An in-depth pathway analysis has been performed to discover how metabolic pathways (glucose, lipid, amino acid, and nucleotide metabolites) are perturbed in different grades of bladder cancer. Glucose metabolism was investigated through different multi-omics analyses in studies on cancer progression. It has been shown in BCa cells, as well as in other tumors [45,46,47,48,49,50,51], that a shift from phosphorylation in mitochondria to aerobic glycolysis occurs, commonly known as the Warburg effect [97,102]. In aerobic conditions, glycolysis is an inefficient way of obtaining energy if compared to oxidative phosphorylation. However, it confers several advantages to cancer cells, such as obtaining intermediates to replenish the anaplerotic metabolism [103,104,105].

Many metabolites and genes involved in the Warburg effect have been detected in BCa metabolomic studies. Tripathi, Conde et al. have confirmed the theory about the over-production of lactate concurrent to progression to highly proliferative stages in BCa [89,90,91,92,93,94,95,96,97]. Petrella et al. [98] observed that alanine excretion was higher for RT4 cells, showing a very clear trend between different grades of malignancy. The pyruvate produced by glycolysis can either be transformed into lactate in the cytosol or enter the mitochondria, where it can be converted to alanine through the transamination reaction. The value of lactate excretion is directly proportional to the degree of glycolysis activity, whereas the degree of alanine excretion can be used as a measure of mitochondrial and oxidative phosphorylation (OxPhos) activities. For this reason, the lactate/alanine ratio is a metabolic measurement of the aerobic/anaerobic balance. Data reported by Loras et al., from T2 BCa cells [27], support a strong down-regulation of OxPhos and the overexpression of glucose metabolic genes (SLCA1, HK2, and RPIA) associated with high levels of lactate. Furthermore, Jin et al. [20] examined the expression of components of the pyruvate dehydrogenase complex (PDC), which is involved in the pyruvate–acetyl-CoA shift in mitochondria. Through microarray analysis, they showed that dihydrolipoyl dehydrogenase (DLD) was significantly reduced in BCa, suggesting that acetyl-CoA levels in BCa are independent from pyruvate.

Under lactate overproduction, several transmembrane transporters are employed to avoid lactic acidosis. Afonso et al. [106] investigated the expression of monocarboxylate transporters (MCTs) involved in intracellular pH regulation. As a representative rate-limiting enzyme, MCT4 transports lactate out of the cell and MCT1 regulates the entry of lactate into tumor cells. The immunohistochemical analysis of MCT1 and MCT4 expression in BCa tumor cells revealed a correlation with poor prognosis. This overexpression has also been found in cancer-associated fibroblasts (CAFs) as the result of a complementary behavior of cells: these findings highlight the role of the tumor microenvironment in sustaining tumor proliferation and invasion [105,106,107,108].

The lipid metabolism also plays a key role in cell motility, cell invasion, and tumor metastasis. It has been suggested that a perturbation in phospholipid metabolism is associated with tumor progression and aggressiveness [89,109,110]. The increase of FA β-oxidation is an energy source in proliferating tumor cells, as discovered in the identification of lower TG levels in patients with BCa compared to healthy controls [89]. Differences in TGs levels between HG and LG BCa lines may reflect the distinct reliability in β-oxidation to generate energy. Decreased levels of myristic, palmitic, and palmitoleic acids have been described in high-grade cell lines. These findings are consistent with the need to obtain energy through FA β-oxidation to continue growing and proliferating [99]. Elevated levels of acetoacetate levels found by Cao et al. [82] in BCa serum samples might be related to the typical increased ketogenesis. Indeed, rapidly proliferating cancer cells overproduce acetyl-CoA through β-oxidation, exceeding its employment in the Krebs cycle. Then, Acetyl-CoA is used in the biosynthesis of ketone bodies (including acetoacetate, b-hydroxybutyrate, and acetone) to replenish the CoA required for further β-oxidation.

Several studies have suggested a potential role of carnitine in cancer progression. High carnitine levels are essential to maintain the transport of acyl groups across the mitochondrial inner membrane when processed in β-oxidation. Jin et al. [20] showed increased excretion of carnitine and acyl-carnitines in the urine samples of BCa patients, consistently with the altered expression of carnitine–palmitoyl–transferase I (CPT1) described in BCa. CPT1 is a key regulator of fatty acid β-oxidation in cells, and the subtype CPT1A is generally overexpressed in BCa cells, presenting a direct relation with tumor cells’ aggressiveness. Conversely, Vantaku et al. [111], in their multi-omics analysis, demonstrated a low expression of subtypes CPT1B in HG-BCa tumors. This finding seemed to be associated with low fatty acid β-oxidation and low acyl carnitine levels in high-grade BCa cells, which has been confirmed in tissue microarrays. Furthermore, induced overexpression of the CPT1B in HG-BCa cells led to a reduced epithelial–mesenchymal transition (EMT) in vitro. This could strengthen the hypothesis that CPT1B plays a crucial role in BCa progression.

## 5. Conclusions

Metabolomics is a promising field in biomarker discovery for BCa. However, there are still some challenges and limitations, as most of the studies are limited to small cohorts without validation through quantitative standardized methods. Metabolite analyses show the aberrant metabolic pathways of BCa, and these findings suggest their potential function as biomarkers for the early detection of BCa. Urine samples are promising in BCa diagnostics, but should be investigated in larger multi-center studies. Several studies have shown that the urine metabolic profile is highly influenced by clinical conditions, genetic background, race, age, sex, lifestyle, diet, and drugs. Multi-omics offers information about the concept of “metabolic inversion” as a signature pathway associated with BCa progression. This aspect could also represent a potential value in clinical outcome prediction. However, further studies to better evaluate metabolism in physiological and pathological conditions are needed.

## Figures and Tables

**Table 1:** Summary of molecular subtypes of non-muscle-invasive bladder cancer.

Refs.	Staging	Differentiation	Marker	Subtypes	Main Genes Mutated	Prognosis
[33]	NMIBC	Luminal	UPKS PPARG, GRHL3, BAMBISPINK1	Class I	FGF3	Good
			UPKs, PPARG. KRT20,GRHL3, BAMBI, SPINK1	Class II	TP53, ERCC2, APOBEC	Poor
		Basal	KRT5, KRT14,KRT15,CD44	Class III	FGF3	Intermediate/Poor
[13]	MIBC	Luminal	KRT20, UPK1A, UPK2, PPARG, GATA3, FOXA1, ESR2	Papillary (LumP)	FGF3, TP53	Good
				Non-specified (LumNS)	ELF3, TP53	Poor
				Unstable (LumU)	PPARG, ERBB2	Intermediate
		Basal	KRT5/6, KRT14, CD44, STAT3	Stroma-Rich	TP53, RB1	Intermediate
				Basal/Squamous (Ba/Sq)		Poor
		Neuronal	CGA, CD56, synaptophysin	Neuroendocrine-like (NE-like)	TP53, RB1	Poor

APOBEC: apolipoprotein B MRNA-editing enzyme catalytic subunit; BAMBI: BMP and activin membrane-bound inhibitor; KRT: cytokeratin; UPK: uroplakin; PPAR: peroxisome proliferation-activated receptor gamma; CGA: glycoprotein hormones, alpha polypeptide; CD: cluster differentiation; ESR: estrogen receptor beta; ELF: E74-like ETS transcription factor; ERBB: erythroblastic oncogene B; ERCC2: excision repair 2, FGF: fibroblast growth factor; FOX: forkhead box; GATA: GATA-binding protein; GRHL: grainyhead-like transcription factor; RB: retinoblastoma transcriptional corepressor; SPINK: serine peptidase inhibitor, Kazal type; STAT: signal transducer activation of transcription; TFIIH: core complex helicase subunit; TP: tumor protein.

**Table 2:** Summary of MS techniques—separation-coupled and NMR coupled.

Technique	Employment	Pros	Cons
GC-MS	Detection and quantification of a wide range of metabolites (volatiles and non-volatiles) without modifications.	High resolution, fit for complex biological samples.	Not effective on thermolabile compounds. Difficulties with the identification of unknown compounds.
LC-MS	Detection and quantification of strongly to slightly polar metabolites.	High sensitivity, good resolution, effective on thermolabile compounds.	Need to reduce volatility and to reduce the potential loss of metabolites.
CE-MS	Detection and quantification of polar metabolites with small sample volumes.	Small volumes with high resolutions.	Complexity when identifying compounds and buffer incompatibility.
NMR-MS	Detection and quantification of monomolecular organic compounds in a large, broad spectrum, using ^1^H, ^13^C, and ^31^P (naturally abundant in biological samples).	Feasible in a wide range of processes for qualitative and quantitative evaluation through a non-biased, fast, and reusable technique.	Cost, and low sensitivity for metabolite detection.

## Supplementary Materials

The following supporting information can be downloaded at: https://www.mdpi.com/article/10.3390/ijms23084173/s1.
