# Supplementary material for: Metabolomic Approaches for Detection and Identification of Biomarkers and Altered Pathways in Bladder Cancer
Source: Int J Mol Sci. 2022 Apr 10;23(8):4173. doi: 10.3390/ijms23084173 (PMC9030452; doi:10.3390/ijms23084173)
Supplement: Supplementary file 1 [file ijms-23-04173-s001.zip › ijms-1637198-supplementary.pdf]

Supplementary Table S1: Summary of BCa Metabolomic studies reviewed

	First author, Year	Methods	Samples	Altered metabolites	Notes	AUC	Altered pathways	Refs
Urine	Pakisanti, 2010	GC/TOFMS<br><br>OPLS-DA	24 BCa (any stage) vs 51 non BCa controls	Senecioic acid<br>2-Butenedioic acid<br>Ribonic acid<br>2,5-Furandicarboxylic acid<br>Sumiki's acid<br>2-Propenoic acid<br>Glycerol<br>Gluconic acid<br>Valerate<br>Fructose<br>Citric acid<br>Ribitol	Decreased			[66]
				Melibiose<br>Uridine<br>L-Valine	Increased			
	Putluri, 2011	PLSDA	83 BCa vs 51 Controls<br><br>Group 1:<br>13 BCa (prior to surgery) vs 13 Controls (age matched with no history of BCa)<br><br>Group 2:<br>28 BCa vs 16 Controls (with prior history of BCa but disease free of benign after treatment)<br><br>Group 3:<br>34 BCa vs 11 Controls(with prior history of BCa but disease free of benign after treatment)<br><br>Group 4:<br>8 BCa vs 11 Controls (age matched with no history of BCa)	Palmitic acid<br>Creatine<br>Histamine<br>Uracil<br>Guanine<br>D−Ribonolactone<br>Pipecolic acid<br>Thymine<br>S−Adenosylmethionine<br>Niacinamide<br>Hippuric acid<br>N12−Acetylspermidine<br>Serine<br>Lysine<br>Spermidine<br>Carnitine<br>Tyrosine<br>3−Hydroxykynurenine<br>Phenylalanine<br>Valine<br>4−Pyridoxic acid<br>Hypoxanthine<br>Kynurenine<br>Histidine<br>Leucine/ Isoleucine	Group 1<br>Group 2<br>Group 3<br>Group 4<br><br>Group 2<br>NMIBC vs MIBC	0.790<br>0.670<br>0.620<br>0.760<br><br><br>0.840		[67]
	Shen, 2015	UPLC-TOF-HRMS<br><br>OPLS-DA	23 BCa (early stages) vs 21 non BCa controls	Nicotinuric acid<br>Aspartateaspartateglycinetriptophan<br>Trehalose	Increased	0.774<br>0.743<br>0.776		[68]
				Glycinecysteinealaninelysine<br>Inosinic acid<br>Ureidosuccinic acid	Decreased	0.834<br>0.720<br>0.752		
	Cheng, 2018	LC-HRMS<br>OPLS-DA	26 NMIBC vs 39 Healthy Controls	Metabolite panel:<br>Dopamine 4-sulfate,<br>mg00/1846z,9z,12z,15z/00,<br>aspartyl-histidine<br>Tyrosyl-methionine		0.838	Fatty acid metabolism Increased<br><br>Fatty acid oxidation	[69]

			43 LG NMIBC vs 43 Healthy controls	Metabolite panel:<br>3-hydroxy-cis-5-tetradecenoylcarnitine<br>6-ketoestriol<br>Beta-cortolone<br>tetrahydrocorticosterone<br>heptylmalonic acid		0.899	Decreased<br><br>Tryptophan metabolism	
			43 LG NMIBC without hematuria vs 37 HG NMIBC without hematuria	Metabolite panel:<br>N-acetyl-4-o-acetylneuraminic acid<br>4-(2-aminophenyl)-2<br>4-dioxobutanoic acid<br>6-keto-decanoylcarnitine<br>3-hydroxydecanoyl carnitine<br>2-hydroxylauroylcarnitine		0.755		
			18 LG NMIBC with hematura vs 69 HG with hematuria	Metabolite panel<br>Indolylacryloylglycine<br>histidinyl-histidine<br>indoleacrylic acid<br>N-acetyl-5-methoxykynuramine<br>l-3-hydroxykynurenine		0.827		
	Jin, 2014	HPLC-MS<br>OPLS-DA	138 BCa vs 121 Healthy controls	Succinate<br>Pyruvate<br>Oxoglutarate<br>Carnitine<br>Phosphoenolpyruvate<br>Trimethyllysine<br>Isovalerylcarnitine<br>Octenoylcarnitine<br>Acetyl-Coa	increased	0.937	Fatty acid oxidation	[20]
				Melatonin<br>Glutarylcarnitine<br>Decanoylcanitine	decreased			
	Loras 2018	UPLC-TOF-MS<br><br>PLS-DA	27/33 BCa vs 10/11 Controls (patient 2 weeks post-TURBT)	Phenylalanine<br>Tryptophan<br>Kynurenine<br>hydroxykynurenine<br>phenylacetylglutamine		0.94	phenylalanine, arginine, proline, and tryptophan	[70]
			BCa vs Patient under surveillance			0.75		
			Controls vs patient under surveillance			0.53		
	Liu, 2018	OPLSA-DA	33 BCa vs 44 age- and sex- matched healthy subjects	Metabolite panel<br>Trans-2-dodecenoylcarnitine<br>serinyl-valine<br>feruloyl-2-hydroxyputrescine<br>3-hydroxynonanoyl carnitine		0.956	Amino acid metabolism and fatty acid oxidation upregulated, phenylalanine metabolism and xenobiotics (e.g., caffeine) metabolism were downregulated	[73]
			15 HG BCa vs 18 LG BCa	Indolylacryloylglycine<br>N_2-galacturonyl-L-lysine<br>aspartyl-glutamate		0.891		
	Pinto, 2021	VOCs headspace solid-phase	53 BCa: 26 Ta/is<br>17 T1<br>10 ≥T2<br>vs 53 Healthy controls	(1S)-1,5-dimethyl-6,8 dioxabicyclo[3.2.1] octane<br>2-butanone<br>2-furaldehyde	Decreased	0.800		[104]

		microextra<br>ction-GC-<br>MS<br>PCA<br>PLS-DA		2-methylbutanal<br>Formaldehyde<br>Hexanal<br>4-heptanone<br>Carvone<br>Piperitone<br>2 unknows				
				1-methylnaphthalene<br>2-methylnonane<br>2-methylnaphthalene<br>2,4-dimethylheptane<br>2,6-dimethylnonane<br>4-methyloctane<br>P-cresol<br>1,2,4-trimethylbenzene<br>4 unknowns	Increased	0.787		
				17 Metabolite set (excluding unknowns)	BCa vs HC<br>Ta/is vs HC<br>T1 vs HC<br>≥T2 vs HC<br>T1 vs Ta/is<br>≥T2 vs Ta/is<br>≥T2 vs T1	0.851<br>0.761<br>0.910<br>0.820<br>0.837<br>0.938<br>0.894		
Serum	Cao, 2012	1H-NMR<br>OPLS-DA	BCa vs Calculi vs Healthy controls	Isoleucine/leucine<br>tyrosine<br>Lactate<br>Glycine,<br>Citrate	Decreased			[84]
				V/ldl<br>Glucose<br>Acetoacetate				
	Lin,2012	LC-MS<br>OPLS-DA	24 BCa vs 24 KC vs 24 Healthy controls	Eicosatrienol<br>Azaprostanoic acid	BCa specific biomarkers	0.980<br>0.977		[85]
				Acetylphenylalanine<br>Methyl hippuric acid	Common genitourinar<br>y biomarkers	0.847<br>0.828		
	Liu, 2020	LC-MS	64 BCa vs 74 KC vs 141 Healthy controls	Homocysteine<br>Thiolactone<br>Acetylcysteine<br>Methionine<br>Sulfoximine<br>9,10,13-trihome<br>Avenoleic acid<br>(10E,12Z)-(9S)-9-<br>Hydroperoxyoctadeca-10<br>12-dienoic acid<br>16-Hydroxy-10-<br>oxohexadecanoic acid	Decreased in cancer groups	0.942		[86]
				9S, 10R-Epoxy-6Z-nonadecene	Increased in cancer groups			
	Bansal,2013	1HNMR<br>OPLS-DA	36 LG BCa vs 31 HG BCa vs 32 Healthy controls	Dimethylamine (DMA)<br>Malonate<br>Lactate<br>Glutamine<br>Histidine<br>Valine		0.910<br>0.905<br>0.897<br>0.891<br>0.899.<br>0878		[87]

	Zhou, 2016	GC-MS-SIM and nontargeted GC-MS<br><br>PLS-DA	53 HG BCa vs 48 age and sex matched Healthy controls	Gluconic acid<br>Xylitol<br>2-keto-gluconic acid<br>Ribose,<br>Arabitol<br>Hypoxanthine<br>Uridine<br>Hypotaurine<br>Kynurenine<br>N-acetylserine	Increased		PPP cycle<br>TCA cycle<br>and fatty acid biosynthesis	[88]
			39 LG BCa vs 48 age and sex matched Healthy Controls	Oleic acid<br>Serine<br>3-indolepropionic acid<br>Glyoxylic acid<br>Glycolic acid<br>Hippuric acid				
			101 HG/LG BCa vs 48 Healthy Controls	Fumaric acid<br>Malic acid<br>Dsaccharic acid	increased	0.863		
	Tan, 2016	UHPLS-QTOFMS	LG BCa vs HG BCa	Inosine<br>N1- acetyl-N2-formyl-5-methoxykynuramine<br>phosphatidylserine		0.961		[89]
	Vantaku, 2019	LC-MS	18 AA BCa VS 54 EA BCa	Taurine<br>Glutamine<br>Glutamate<br>Aspartate<br>Serine<br>Hypoxanthine<br>Uric acid	Increased in AA		one-carbon, tryptophan and nucleotide metabolisms (e.g. purine catabolic pathway)	[90]
Tissue	Putluri, 2011	LC-MS	27 benign adiacent vs 31 BCa vs 25 matched pairs	Serine<br>Asparagine<br>Valine<br>Tryptophan<br>Phenylalanine<br>Histidine	Increased			[67]
	Tripathi, 2013	1H-HRMS-NMR –<br>GC-MS crossvalidation<br><br>PCA and PLS-DA	17 ta-t1 BCa tissues vs 16 T2 BCa tissues vs 26 benign/healthy tissues vs	branched-chain amino acids (BCAAs), Lactate<br>Alanine<br>Ace<br>Glutamic acid<br>GSH<br>Glutamine<br>Aspartate<br>Cre<br>ChoCC<br>Taurine<br>myo-Inositol<br>Phenylalanine,<br>Tyrosine	Increased in BCa vs healthy<br><br>No clear segregation observed between ta-t1 and t2	0.970	aerobic glycolysis (Warburg effect)<br>TCA cycle anaplerosis	[91]
	Piyarathna, 2017	LC-MS	126 BCas vs 39 benign adiacent/normal	phosphocholines<br>phosphatidylethanolamines<br>plasmenyl Pes	Increased		cyclooxygenase-2 pathways implicated in tumor growth and metastasis	[92]
				Triglyceride	Decreased			

	Yang, 2019	LC-HRMS UPLC<br><br>PCA	48 samples collected in 4 groups: Pre-gemcitabine normal, pre-gemcitabine BCa, post-gemcitabine normal, and post-gemcitabine BCa	Bilirubin	Decreased in BCa tissues pre-gemcitabine and insignificant level after gemcitabine		glutathione, purine and thiamine metabolism	[93]
				Retinal	In T2 stage BCa vs normal Decreased<br><br>And insignificant difference after gemcitabine			
Cell lines	Dettmer, 2013	LC-QTOFMS PCA (Principal Component Analysis	Cluster 1 HCC and 5 other cell lines of different origin (JB2 BCa LINE)<br>Cluster 2: Renal Cell Carcinoma and BCa RT4<br>CLUSTER 3: primary hepatocytes<br>Cluster 4: Mammary carcinoma lines and THE T47D melanoma cell line<br>Cluster 5: SW 480 AND SW620 colon carcinoma lines	dipeptides Val-Leu and Leu-Leu	Increased in RT4 (cluster 2)<br><br>Decreased in JB2 (Cluster 1)		carrier-mediated uptake or extracellular hydrolysis followed by uptake of amino residues by amino acid transporters . pathways related to increased use of di and tripeptides ad substrates of increased bioenergetic and biosintetic demand in cancer cells	[98]
	Conde, 2015	(1H-NMR) and spectra analysis	RT4 (less invasive) vs TCCSUP cell lines (more invasive)	glucose	Decreased in both		metabolism of pyruvate	[99]
				Pyruvate	Decreased in TCCSUP			
				Alanine	Increased in TCSSUP			
	Petrella, 2020	1H-NMR spectroscopy exometabolome analysis<br><br>OPLS-DA	5637 vs RT112 vs RT4 cell lines	Glucose<br>Glutamine<br>serine<br>BCAAs<br>arginine	Decreased in each one line(consumed)<br>Glucose: similar		Glycolysis is the Most Active Metabolic Pathway in 5637 Cells<br><br>RT Cells Show an Active Oxidative Metabolism	[100]
				Alanine<br>Lactate<br>Pyruvate<br>formate	Increased in each one line (excreted)<br>Lactate: 5637/RT112 > RT4<br><br>Alanine: RT4 > RT112 > 5637			

	Rodrigues, 2019	GC-MS endometabolome analysis<br><br>PCA PLS-DA	J82 vs 5637 Cell lines	Glycine<br>Myristic acid<br>Palmitic acid<br>palmitoleic acid	Decreased in J82	0.960<br>1.000<br>1.000<br>1.000	Ammonia recycling, fatty acid biosynthesis, and methionine and glutamate metabolisms	[101]
				Leucine<br>Methionine<br>Valine<br>Aspartic acid	Increased in J82	0.880<br>0.920<br>0.920<br>0.960		
	Iliou, 2020	1H-NMR spectroscopy<br><br>PCA<br>PLS-DA	RT4 (grade I)<br>RT112 (grade II)<br>T24 (grade III) TCCSUP (grade IV)	Myo-inositol<br>Creatine phosphate<br>Histidine<br>Malate<br>Succinate,<br>Acetate<br>AMP<br>ADP<br>ATP<br>GTP<br>UMP<br>Oxypurinol	Increased in T24<br><br>Decreased In RT4 and TCCSUP		Metabolic inversion	[102]
				Uracil<br>Propylene glycol	Decreased in T24 and TCCSUP			
				Tryptophan<br>Formate<br>Uracil<br>Adenine<br>Propylene glycol<br>Choline	Ratio decreased in grade III (T24) compared to grade I (RT4)			
	Rodrigues, 2018	HSSPME/GC–MS VOCs extraction<br><br>OPLS-DA	Scaber<br>J82<br>5637<br>SV-HUC-1 (normal)	2-pentadecanone<br>A-terpineol<br>2-methylbutan-2-ol<br>1-phenylethanol<br>2-phenylpropan-2-ol<br>2-hydroxy-2-methyl-1-phenylpropan-1-one	Scaber vs SV-HUC-1 Ph7	1.000	synthesis of fatty acids for membrane formation or β-oxidation of fatty acids for energy production, amino acids metabolism, oxidative stress and inflammation process	[103]
				Benzoic acid<br>2-methyl-2-heptanol<br>1-butanol<br>A-methylstyrene<br>unknown 25	Scaber vs SV-HUC-1 Ph2	1.000		
				4-methylbenzaldehyde<br>cyclohexanone<br>2-pentadecanone<br>2-phenylpropan-2-ol<br>phenol<br>dodecane<br>4-methylheptan2-one<br>γ-dodecalactone<br>unknown 24	J82 vs SV-HUC-1	1.000		
				1-phenylethanol<br>4-methylnonane<br>dodecane<br>γ-dodecalactone<br>unknowns 10 and 24	5637 vs SV-HUC-1	1.000		

				benzaldehyde<br>2-nonanone<br>4-methylheptan-2-one<br>dodecane 2<br>3-dimethylhexane<br>hexadecane<br>tetradecane	Increased in BCa lines medium vs normal line mediun	1.000		
				2-phenylpropanol<br>isopentanol	Decreased in BCa cell lines medium vs normal line medium	1.000		
				acetophenone<br>α-terpineol<br>nonanal<br>2-phenylpropan-2-ol<br>4-methylnonane<br>6-methylheptan-2-one	Decreased in J82 and Scaber vs 5637 medium	1.000		
				Dodecanol<br>dodecanal<br>γ-nonalactone	Increased in J82 and Scaber vs 5637 medium	1.000		
				1-phenylethanol<br>α-terpineol<br>(2E,4E)-Deca-2,4-dienal<br>Benzaldehyde<br>Benzoic acid<br>Geranylacetone<br>2-pentadecanone<br>γ-nonalactone	Increased in J82 vs Scaber	1.000		
				Phenol<br>Nonanal<br>n-butyl acetate<br>acetophenone<br>Cyclohexanone<br>Naphthalene	Decreased in J82 vs 5637 medium	1.000		
